# Heterogeneous responses of dorsal root ganglion neurons in neuropathies induced by peripheral nerve trauma and the antiretroviral drug stavudine

**DOI:** 10.1002/ejp.541

**Published:** 2014-07-29

**Authors:** EK Boateng, A Novejarque, T Pheby, ASC Rice, W Huang

**Affiliations:** 1 Pain Research Group, Department of Surgery and Cancer, Faculty of Medicine, Imperial College LondonUK; 2 Institute of Medical Sciences, University of AberdeenUK

## Abstract

**Background:**

Heterogeneity is increasingly recognized in clinical presentation of neuropathic pain (NP), but less often recognized in animal models. Neurochemical dysregulation in rodent dorsal root ganglia (DRG) is associated with peripheral nerve trauma, but poorly studied in non-traumatic NP conditions.

**Methods:**

This study aimed to investigate the temporal expressions of activating transcription factor-3 (ATF-3), growth-associated protein-43 (GAP-43), neuropeptide Y (NPY) and galanin in traumatic and non-traumatic rat models of neuropathies associated with NP. Expressions of these markers were examined in the DRG at different time points following tibial nerve transection (TNT) injury and antiretroviral drug stavudine (d4T) administration using immunohistochemistry. The development of sensory gain following these insults was assessed by measuring limb withdrawal to a punctate mechanical stimulus.

**Results:**

Both TNT-injured and d4T-treated rats developed hindpaw mechanical hypersensitivity. Robust expressions of ATF-3, GAP-43, NPY and galanin in both small- and large-sized L5 DRG neurons were observed in the DRG from TNT-injured rats. In contrast, d4T-treated rats did not exhibit any significant neurochemical changes in the DRG.

**Conclusions:**

Taken together, the results suggest that ATF-3, GAP-43, NPY and galanin are likely indicators of nerve trauma-associated processes and not generic markers for NP. These experiments also demonstrate distinct expression patterns of neurochemical markers in the DRG and emphasize the mechanistic difference between nerve trauma and antiretroviral drug-associated NP.

## 1. Introduction

Rodent models of nerve trauma are conventionally used to elucidate neuropathic pain mechanisms and to develop novel drugs. Although physical trauma to peripheral nerves is one insult that may elicit neuropathic pain in humans, the most widespread causes arise from various aetiologies (Jensen, 2001), and animal models are evolving to reflect that fact (Rice, 2010). Heterogeneity of presentations and mechanisms of neuropathic pain has been increasingly appreciated in the clinical domain (Baron et al., 2012), but less so preclinically.

About 40% of patients whose HIV infection is otherwise well controlled by antiretroviral therapies (ART) suffer intractable neuropathic pain, and one of the major factors underlying such a complication is a neurotoxicity induced by certain ART drugs (Cherry et al., 2012). Although toxic ART drugs are being phased out, it will take considerable time to eliminate the use of the cost-effective ART stavudine (d4T) in many resource-limited settings. Thus, understanding the pathophysiology of ART-associated neuropathies is a vital part of drug development. We, and others, have shown that toxic ART-treated rats develop bilateral hindpaw mechanical hypersensitivity and exhibit complex pain-related behaviours (Joseph et al., 2004; Huang et al., 2013) similar to those observed in nerve trauma models (Hasnie et al., 2007). Moreover, these animals had reduced intradermal nerve fibre density, thus providing evidence of nerve damage (Wallace et al., 2007; Huang et al., 2013).

**What's already known about this topic?**

- We know that there are differences in gene expression and microgliosis in the dorsal root ganglia between traumatic and non-traumatic rodent models of neuropathic pain.

**What does this study add?**

- This study further elucidates the distinct differences in the neurochemical responses to nerve damage in the dorsal root ganglia in the two different models, highlighting the heterogeneity of mechanisms underlying neuropathic pain from different aetiologies.

Recently, differences in the dorsal root ganglion (DRG) gene expression and spinal microgliosis have been revealed between rodent models of peripheral nerve trauma and chemotherapy- or ART-induced peripheral neuropathy (Maratou et al., 2009; Zheng et al., 2011a; Blackbeard et al., 2012).

Following nerve trauma, a number of neurochemical changes that have been linked to neuropathic pain mechanisms (Hulse et al., 2012) occur within DRG neurons, including the neuronal injury marker activating transcription factor-3 (ATF-3) (Tsujino et al., 2000), neuropeptide Y (NPY) (Ma and Bisby, 1998), galanin (Hokfelt et al., 1987) and growth-associated protein-43 (GAP-43) (Sommervaille et al., 1991). However, these studies are difficult to compare due to different methodologies, species and strains used and also because of variability of nerve injuries. Moreover, these markers were mostly examined at specific single time points post nerve trauma; thus, the temporal expression profiles encompassing both the acute and chronic stages of nerve injury as well as phenotypic cell effects are not comprehensively documented. Importantly, these markers have not been fully investigated in non-traumatic neuropathic pain models. Only recently, we showed DRG expression of ATF-3 and NPY at 7 and 21 days following d4T treatment (Huang et al., 2013).

In this study, we hypothesized that the temporal expressions of neurochemical markers ATF-3, NPY, galanin and GAP-43 in the d4T model are different to those observed following tibial nerve transection (TNT) injury in rats. The rationale for choosing ATF-3, GAP-43, NPY and galanin is described in the Supporting Information Appendix S1.

## 2. Methods

### 2.1 Ethical statement

All experiments conformed to the UK Animal (Scientific Procedures) Act 1986 and were conducted under the authority of Home Office Project License PPL70/7162. We followed International Association for the Study of Pain guidelines for animal use and care and ARRIVE guidelines for data reporting (Zimmermann, 1983; Kilkenny et al., 2011).

### 2.2 Animal maintenance

Adult male Wistar rats (250–300 g; Charles River, Margate, Kent, UK) were housed in temperature-controlled individually ventilated cages (21 °C, 1500 cm^2^ floor area, four per cage), maintained on a 12:12 h light–dark cycle and provided with normal rat chow food (Special Diet Services, Essex, UK) and tap water *ad libitum*. Corncob soft bedding was used (Lillico Biotechnology, Hookwood, Surrey, UK). Animals were allowed to acclimatize in their housing environment for at least 48 h following arrival, and all behavioural experiments were performed during the light circle (7 a.m.–7 p.m.).

### 2.3 Study design

All treatments were assigned per cage and animals were grouped based on survival times following hindpaw mechanical sensory testing. TNT/sham-operated animals (*n* = 36) were divided into the following groups: TNT day 1 (*n* = 3), sham day 1 (*n* = 4); TNT day 7 (*n* = 3), sham day 7 (*n* = 3); TNT day 14 (*n* = 3), sham day 14 (*n* = 4); TNT day 28 (*n* = 4), sham day 28 (*n* = 4); TNT day 43 (*n* = 4), sham day 43 (*n* = 4). Animals treated with d4T/saline were divided into the following groups: d4T day 7 (*n* = 4), saline day 7 (*n* = 4; d4T day 21 (*n* = 4), saline day 21 (*n* = 4); d4T day 43 (*n* = 4), saline day 43 (*n* = 4). Naïve animals (*n* = 4) did not undergo any sensory testing assessment and were only used as histological controls. Necessary steps, e.g., major domains of good laboratory practice (Macleod et al., 2009), were taken to minimize the impact of experimental bias (Supporting Information Table S1) on both hindpaw mechanical sensory testing and immunohistochemical analysis.

### 2.4 Hindpaw withdrawal thresholds

Paw withdrawal thresholds (PWTs) to punctate mechanical stimuli was measured using an electronic von Frey device (Somedic, Horby, Sweden). Animals were placed into a Plexiglas clear box (23 × 18 × 14 cm, Linton Instrument, Norfolk, UK) with a 0.8 cm in diameter mesh floor, and allowed to acclimatize (approximately 10–15 min). The calibrated force transducer (0.5 mm^2^ diameter tip) was applied at a rate of 8–15 g/s to the mid-plantar surface of each hindpaw until an active limb withdrawal response was observed. A mean PWT was then calculated from a set of five applications.

### 2.5 Immunohistochemistry

Naïve rats were immediately sacrificed and nerve-damaged rats were sacrificed shortly after recording of hindpaw mechanical responses with an intraperitoneal injection of pentobarbital (300 mg/kg; Animal Care Ltd., York, UK) and transcardially perfused with 200 mL of heparinized 0.9% saline followed by 300 mL of 4% paraformaldehyde solution (pH 7.4) in 0.1 M phosphate buffer. The L5 DRGs were harvested and post-fixed in 4% paraformaldehyde for 2–4 h and transferred to a 30% sucrose solution in 0.01 M phosphate-buffered saline (PBS) for 72 h. DRGs were then embedded in OCT mounting medium (VWR, Lutterworth, UK). Serial cryostat sections (10 μm) were thaw mounted on Superfrost® slides (VWR) allocated for individual animals. Sections were rinsed with PBS (3 × 10 min) before incubation with PBS Triton (0.01 M PBS, 0.2% Triton and 0.1% sodium azide) containing 10% normal goat serum for 1 h at room temperature. Immunohistochemical reactions were conducted overnight using the following primary antibodies diluted in PBS Triton: rabbit anti-ATF-3 (1:200; Santa Cruz Biotechnology, Dallas, TX, USA); rabbit anti-galanin (1:4000; Peninsula Laboratories, San Carlos, CA, USA); rabbit anti-NPY (1:1000; Peninsula Laboratories); rabbit anti-GAP-43 (1:1000; Chemicon, Watford, UK). For the identification of cellular phenotype, sections were double labelled with mouse anti-peripherin (1:100; Santa Cruz Biotechnology or 1:1000; Abcam, Cambridge, UK) or mouse anti-neurofilament 200 (1:1000; Sigma, Dorset, UK). Following incubation of primary antisera, slides were rinsed (3 × 10 min) with PBS and incubated using the following secondary bodies in PBS Triton: goat anti-mouse Alexa flour 568 (1:400; Invitrogen, Paisley, UK) and goat anti-rabbit Alexa flour 488 (1:400; Invitrogen). Then sections were washed in PBS and incubated with 2 μg/mL of Hoechst 33342 (Sigma) diluted in distilled water for 2 min, and coverslipped with PBS/glycerol (1:8). Negative controls were carried out for each primary antibody used by omission of primary antisera.

### 2.6 Image analysis and quantification

Fluorescent images were visualized using a fluorescence microscope (Leica, Linford Wood, Bucks, UK), equipped with appropriate filter blocks and captured at consistent exposure times using a charge-coupled device camera (Model 3CCD c5810; Hamamatsu, Hamamatsu City, Japan). Images were analysed using QWin v.3.0 software (Leica). All images were taken at ×10 objective magnification. For each animal a total of three serial sections (at least 150 μm apart) representing the beginning, middle and end sections of the DRG were used to take images for analysis. These three sections were comparable between animals. Monochromatic grey scale images were loaded onto QWin, and a binary overlay was created based on the pixel intensity immunoreactive cells. The threshold for immunoreactivity was determined for each section when there was a clear demarcation point between background and immunoreactivity. Background was determined by selecting an area of the DRG where immunolabelling was not present, and this value was subtracted from the threshold level for immunoreactivity. A binary overlay was constructed based on the intensity and number of pixels of each image, to identify immunoreactive cells. The total number of peripherin/NF-200 immunoreactive neurons expressing immunoreactivity for ATF-3, GAP-43, NPY and galanin was determined and expressed as a percentage of the total number of peripherin/NF-200 immunoreactive neurons for each animal. A mean percentage was then determined for each group. Parallel colour images were taken with nuclear marker Hoechst 33342 and immunoreactive cells cross-checked so that only cells with a demonstrable nucleus were analysed.

### 2.7 Statistical analysis

Sigmastat version 3.5 (Systat Software, Erkrath, Germany) was used to determine statistically significant differences throughout the study. *p* < 0.05 was considered statistically significant. To compare mean PWT at baseline and at each subsequent time point, a Student's paired *t*-test was performed. To compare the temporal profiles of neurochemical markers a one-way analysis of variance (ANOVA) with Holm–Sidak post hoc was performed. A two-way ANOVA followed by Holm–Sidak post hoc test was used to analyse intervention effects (nerve-damaged rats vs. control) and cell size effects (NF-200 vs. peripherin) at each time point. All data are represented as mean ± standard error of the mean.

## 3. Results

### 3.1 Exclusions

For the TNT experiments, two rats were excluded (at day 1 and day 14 post-injury) due to incorrect nerve injury that was confirmed at post-mortem. No d4T rats were excluded.

### 3.2 Temporal development of hindpaw mechanical hypersensitivity following TNT injury or d4T treatment

**Figure 1:**
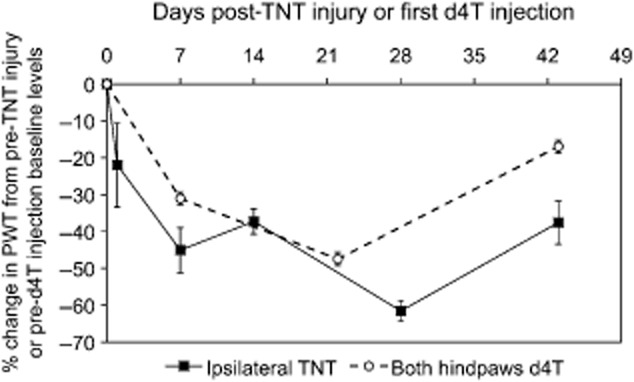
Mean percentage change in paw withdrawal threshold (PWT) from baseline at various time points post tibial nerve transection (TNT) injury and d4T treatment in response to punctate mechanical stimulation. PWT data regarding both hindpaws of d4T rats were pooled since both sets of data were identical.

The temporal effects of TNT injury on the percentage decreases in ipsilateral PWTs from baseline were comparable with that observed in d4T-treated rats (Fig. 1). The time course of TNT and d4T-associated mechanical hypersensitivity can be observed in Supporting Information Figs. S1 and S2. Percentage changes in d4T-treated rats were averaged from both hindpaws due to similar degrees of bilateral mechanical hypersensitivity. In the acute stages of nerve trauma, day 1 post-TNT injury, rats exhibited a 22 ± 11.4% decrease in ipsilateral PWTs. The decrease in PWTs increased further at day 7 post-TNT injury to 45.1 ± 6.1%, while d4T-treated rats at 7 days post the first injection demonstrated a 31.0 ± 1.7% reduction in PWTs. At day 14 post-TNT injury, rats exhibited a 37.3 ± 3.4% decrease in ipsilateral PWTs. At day 28 post-TNT injury, the decrease in ipsilateral PWTs peaked at 61.1 ± 2.9% while at 22 days post the first d4T injection, the decrease in PWTs peaked at 47.5 ± 1.8%. At the longest experimental time point of 43 days, TNT-injured rats demonstrated a decrease in ipsilateral PWTs of 37.6 ± 5.8% while in d4T-treated rats, PWTs decreased by 17 ± 1.8%. Mechanical hypersensitivity was not observed in sham animals throughout the study.

### 3.3 ATF-3 expression

**Figure 2:**
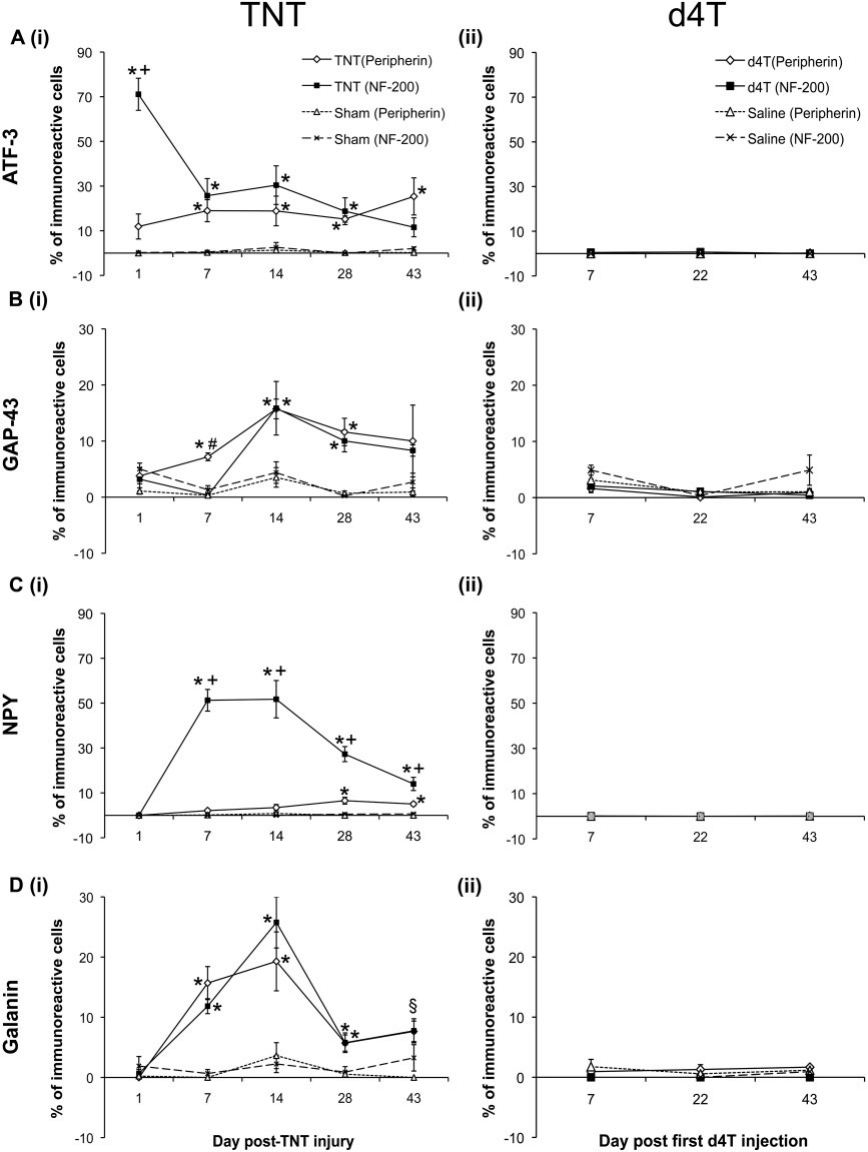
Mean percentage of total NF-200 and peripherin cells immunoreactive for (A) activating transcription factor-3 (ATF-3), (B) growth-associated protein-43 (GAP-43), (C) neuropeptide Y (NPY) and (D) galanin in (i) ipsilateral L5 dorsal root ganglia (DRGs) of tibial nerve transection (TNT) and sham animals; and (ii) the left L5 DRGs of d4T-injected animals. Data are presented as mean ± standard error of the mean. Statistical test at each time point was conducted using a two-way analysis of variance followed by Holm–Sidak post hoc analysis: *p < 0.05 versus respective sham; ^+^*p* < 0.05 versus peripherin-immunoreactive neurons; ^#^*p* < 0.05 versus NF-200-immunoreactive neurons. ^§^*p* < 0.05 TNT (peripherin) versus sham (peripherin).

**Figure 3:**
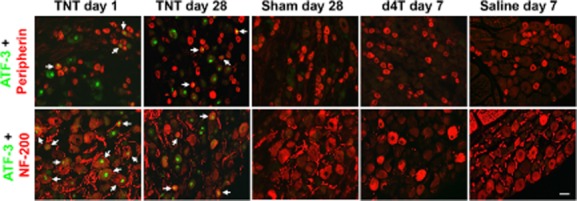
Representative images of activating transcription factor-3 (ATF-3) immunoreactivity. The left three columns are for ATF-3 expression in ipsilateral L5 dorsal root ganglia (DRGs) at 1 and 28 days post tibial nerve transection (TNT) injury and at 28 days post sham surgery. The right two columns are for ATF-3 expression in the left L5 DRGs of d4T/saline-treated rats at 7 days post first injection. Sections were co-labelled with peripherin (top row) and NF-200 (bottom row) to identify ATF-3 immunoreactivity in distinct populations of DRG neurons. Images were captured at ×20 objective magnification. Arrows indicate co-localization of immunolabelling. Scale bar = 50 μm.

Consistent with the literature (Tsujino et al., 2000), ATF-3 immunoreactivity was not evident in DRGs harvested from naïve rats (Supporting Information Table S2). TNT injury was associated with significant ATF-3 up-regulation in both NF-200 and peripherin cell populations when compared with sham rats [*p* < 0.05; Fig. 2A(i); Fig. 3] except at 1 day post-injury in peripherin-immunoreactive cells, and 43 days post-injury in NF-200-immunoreactive cells. ATF-3 immunoreactivity was nuclear (Fig. 3). A significant temporal profile effect was observed in NF-200-immunoreactive cells in TNT-injured rats (*p* < 0.05), which was particularly evident at 1 day after nerve injury, but not in peripherin-immunoreactive cells.

Following d4T treatment, ATF-3 immunoreactivity was observed in very few NF-200 and peripherin-immunoreactive cells. Mean percentage ATF-3 immunoreactivity in NF-200 and peripherin cells was <1% across all time points. No significant differences were observed between percentage ATF-3 immunoreactivity in NF-200 and peripherin-immunoreactive cells at various time points examined and no temporal profile effects were observed [Fig. 2A(ii); Fig. 3].

### 3.4 GAP-43 expression

**Figure 4:**
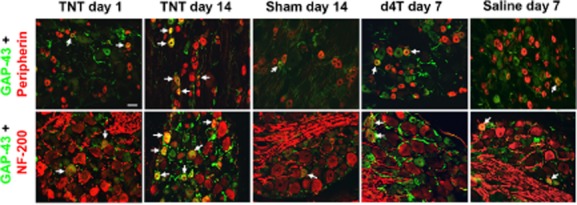
Representative images of growth-associated protein-43 (GAP-43) immunoreactivity. The left three columns are for GAP-43 expression in ipsilateral L5 dorsal root ganglia (DRGs) at 1 and 14 days post tibial nerve transection (TNT) injury and at 14 days post sham surgery. The right two columns are for GAP-43 expression in the left L5 DRGs of d4T/saline-treated rats at 7 days post first injection. Sections were co-labelled with peripherin (top row) and NF-200 (bottom row) to identify GAP-43 immunoreactivity in distinct populations of DRG neurons. Images were captured at ×20 objective magnification. Arrows indicate co-localization of immunolabelling. Scale bar = 50 μm.

GAP-43 immunoreactivity in naïve DRGs was only observed in few NF-200-immunoreactive cells (<2%; Supporting Information Table S2) and not in the peripherin-immunoreactive population. GAP-43 immunoreactivity was cytoplasmic in nature and was observed in both NF-200 and peripherin DRG cell populations, and additionally observed in axons (Fig. 4). TNT injury was associated with significant GAP-43 up-regulation in both NF-200 and peripherin-immunoreactive cells at different time points post-injury compared with sham-operated rats [*p* < 0.05; Fig. 2B(i); Fig. 4]. Significant temporal profile effects were evident in the NF-200 (*p* < 0.05) but not the peripherin-characterized cell populations.

Following d4T treatment, GAP-43 immunoreactivity was observed in few NF-200 and peripherin-immunoreactive cells. Mean percentage GAP-43 immunoreactivity in NF-200 and peripherin-characterized cells was <5% across all time points and was not significantly different to that observed in respective saline-treated rats. No significant differences in GAP-43 immunoreactivity were observed between NF-200 and peripherin-immunoreactive cell populations at various time points examined. A significant temporal profile of GAP-43 immunoreactivity was observed in the peripherin-immunoreactive cells of d4T-treated rats and in the NF-200-immunoreactive cells of saline-treated rats [Fig. 2B(ii); Fig. 4].

### 3.5 NPY expression

NPY immunoreactivity in naïve DRGs was only observed in few NF-200-immunoreactive cells (<0.3%; Supporting Information Table S2) and not in peripherin-immunoreactive cells. NPY immunoreactivity was largely cytoplasmic, although some immunoreactivity was observed in axons (Supporting Information Fig. S3). TNT injury was associated with robust NPY immunoreactivity, particularly in the NF-200-immunoreactive cells [Fig. 2C(i); Supporting Information Fig. S3]. Significant temporal profiles were observed in both peripherin and NF-200-immunoreactive cells [*p* < 0.05; Fig. 2C(i); Supporting Information Fig. S3].

Following the administration of d4T to rats, NPY immunoreactivity was observed in few NF-200 and peripherin-immunoreactive cells. Mean percentage NPY immunoreactivity in NF-200 and peripherin-immunoreactive cells was <0.5% across all time points. No significant differences were observed between percentage NPY immunoreactivity in NF-200 and peripherin-immunoreactive cells at various time points examined and no temporal profile effects were observed [*p* > 0.05; Fig. 4C(ii); Supporting Information Fig. S3].

### 3.6 Galanin expression

Galanin immunohistochemistry in naïve DRGs was only observed in few peripherin-immunoreactive cells (<0.6%, Supporting Information Table S2) and not in NF-200-immunoreactive cells. Galanin exhibited a diffuse granule-like cytoplasmic immunoreactivity in both NF-200 and peripherin-immunoreactive cells (Supporting Information Fig. S4). TNT injury was associated with significant galanin immunoreactivity in both NF-200 and peripherin-immunoreactive cells when compared with respective sham rats at particular time points post-injury, and both cell populations demonstrated dynamic and similar significant temporal profiles [*p* < 0.05; Fig. 2D(i); Supporting Information Fig. S4].

Following the administration of d4T to rats, galanin immunoreactivity was observed in few NF-200 and peripherin-immunoreactive cells. Mean percentage galanin immunoreactivity in NF-200 and peripherin-characterized cells was <2% across all time points. No significant differences were observed between percentage galanin immunoreactivity in NF-200 and peripherin-immunoreactive cells at various time points examined and no temporal profile effects were observed [*p* > 0.05; Fig. 2D(ii); Supporting Information Fig. S4].

## 4. Discussion

Here, we revealed distinct differences in DRG neurochemical response following traumatic TNT injury and systemic exposure to the antiretroviral drug d4T in adult rats. These models have fundamental differences regarding the nature of the nerve damage, yet are both associated with neuropathic pain clinically and with features suggestive of neuropathic pain in animals (Andrews et al., 2012; Huang et al., 2013). Crucially, in both animal models, similar degrees of hindpaw mechanical hypersensitivity were documented. The onset of mechanical hypersensitivity in TNT animals was comparable with previous reports 2–3 weeks following injury lasting for at least 2 months (Andrews et al., 2012). Similarly, in d4T-treated animals, mechanical hypersensitivity was consistent with observations from animal studies using the neurotoxic antiretroviral drug zalcitabine (ddC) (Wallace et al., 2007).

Experimental peripheral nerve injury induces several neurochemical changes in the DRGs that may play a role in abnormal pain states. Our study is the first to examine such changes following TNT injury using immunohistochemical analysis and additionally compared with the d4T-associated neuropathy model. Following TNT injury, ATF-3 was significantly up-regulated in the ipsilateral L5 DRG in both peripherin and NF-200 DRG neurons at all time points compared with sham controls. At day 1 post-TNT injury, ATF-3 expression was significantly greater in large diameter NF-200 expressing cells (71%) compared with the smaller sized peripherin-expressing cells (25%). This may be a reflection on the differing degrees of injury sustained between small- and large-sized neurons or different responses of these neuronal types following TNT injury. In certain types of nerve trauma such as chronic constriction injury, large myelinated fibres incur greater levels of damage and degeneration than unmyelinated fibres (Basbaum et al., 1991), although such changes were observed at 10–14 days post-injury. The biological role of ATF-3 is not entirely clear. Its up-regulation may be indicative of other processes such as nerve regeneration (Seijffers et al., 2007).

The increased ATF-3 immunoreactivity in the DRG after nerve injury is also associated with dysregulated neuropeptide expression, particularly NPY and galanin. From at least day 7 following TNT injury, NPY was up-regulated primarily in NF-200-immunoreactive cells (peak at day 14 post-injury ∼51%), but also in a modest percentage of peripherin cells (1–6.5%). The cell size effect and magnitude of immunoreactivity correlates with previous studies (Ma and Bisby, 1997). The exact role of NPY is unclear, but evidence has implicated NPY in antinociception (Intondi et al., 2008). The significant temporal increase in NPY immunoreactivity in small-sized peripherin-immunoreactive cells seen here may therefore represent a compensatory mechanism to reduce excessive nociception related to neuropathic pain processes.

Galanin exhibited a similar temporal expression patterns between peripherin and NF-200-immunoreactive DRG cells after TNT injury in this study. Pharmacological studies have shown galanin to be antinociceptive in naïve rats, although at low doses (Wiesenfeld-Hallin et al., 1988). Galanin deletion impairs the ability of peripheral nerves to regenerate following sciatic nerve crush and loss-of-function galanin mutations are associated with increased apoptosis and a loss of DRG neurons (Holmes et al., 2000), therefore galanin may regulate nerve regeneration and neuroprotective processes.

Nerve regeneration following nerve trauma has been associated with increased GAP-43 immunoreactivity in the DRGs (Sommervaille et al., 1991). TNT injury initiated GAP-43 up-regulation, with similar temporal profile expressions between peripherin and NF-200 cells. A cell size effect was observed at day 7 post-injury, which corroborates with previous studies demonstrating GAP-43 mRNA up-regulation in initially small DRG neurons before a subsequent increase in large DRG neurons after nerve trauma (Wiese et al., 1992). GAP-43 modulates the formation of new neuronal connections and may be involved in the guidance of growing axons endings (Benowitz and Routtenberg, 1997). GAP-43 expression may also be related to the interruption of axonal transport since its inhibition can induce immunoreactivity identical to nerve crush injury (Woolf et al., 1990).

Although there appears to be an associative trend, our investigations revealed no correlations between the percentage immunoreactivity of investigated neurochemical markers and the degree of pain-related mechanical hypersensitivity following TNT injury. This result concurs with previously reported observations regarding ATF-3 (Shortland et al., 2006) and GAP-43 (Jaken et al., 2011).

The neurochemical dysregulation observed following TNT injury was not apparent in the DRGs harvested from d4T-treated rats, which is in agreement with our previous findings also demonstrating a lack of ATF-3, NPY and galanin immunoreactivity in the DRGs of rats at 21 days after systemic treatment with the ddC (Wallace et al., 2007). In contrast to the d4T model, TNT-injured rats demonstrated an apparent DRG neurochemical response that manifested in the expression of ATF-3, NPY, galanin and GAP-43; all findings consistent with literature reports. Our results suggest these neurochemical markers are likely indicators of nerve trauma-associated processes and not specific generic biomarkers of neuropathic pain mechanisms.

The differences in neurochemical marker expression could reflect differences in other sensory modalities such as cold or thermal. There is evidence supporting thermal hypersensitivity in ddC-treated rats (Joseph et al., 2004), while other studies regard thermal hypersensitivity to not be a consequence of ddC (Wallace et al., 2007) or d4T treatment (Renn et al., 2011). This inconsistency makes this a less reliable measurement of pain-related behaviour.

The lack of neurochemical marker expression in d4T-treated rats could be linked to apoptotic DRG-related neuronal death. The apoptotic marker-activated caspase-3 is present in the DRGs of nerve trauma-injured rats (Sekiguchi et al., 2009), at a time when neurochemical markers such as that observed in our study is also present. Therefore, it is unlikely apoptosis is the cause of the lack of neurochemical marker expression in d4T-treated rats.

Distinct responses to nerve damage between these two models are also observed at gene level within the DRGs (Maratou et al., 2009) and with regard to spinal microglia infiltration, which is relatively modest in d4T/ddC-treated rats, compared with that in rats with nerve trauma (Zheng et al., 2011a; Blackbeard et al., 2012; Huang et al., 2013).

Our results also indicate fundamental mechanistic heterogeneity in the manifestation of neuropathic pain between traumatic and non-traumatic nerve damage. The pathological processes responsible for d4T/ddC-associated neuropathy are likely linked to mitochondrial dysfunction (Payne et al., 2011; Huang et al., 2013), a feature perhaps less relevant in nerve trauma. Such mitochondrial dysfunction is supported via experimental evidence documenting altered calcium homeostasis (Joseph et al., 2004) and activation of pro-apoptotic caspase pathways (Joseph and Levine, 2004). Oxidative stress may play a role, and the removal of reactive oxygen species by scavengers is reported to reverse mechanical hypersensitivity in ddC-treated rats (Zheng et al., 2011b). The lack of ATF-3 and NPY immunoreactivity in the DRG of d4T-treated rats parallels our recent study, which has additionally demonstrated normal CGRP and IB4 expression following d4T administration (Huang et al., 2013). These observations, in conjunction with studies demonstrating a clear neurotoxicity involving retraction of nerve fibres from paw skin (Wallace et al., 2007; Huang et al., 2013), is suggestive of a mechanism not involving the DRGs, in contrast to nerve trauma models where many neurochemical changes occur within the DRGs. However, reports of increased immunoreactivity of pronociceptive macrophage chemoattractant CCL2 (Wallace et al., 2007) and lymphocyte chemoattractant CXCL12 (Bhangoo et al., 2007) in the DRGs of ddC-treated rats indicates involvement of an inflammatory mechanism and perhaps does not entirely rule out pathological processes within the DRGs with this type of injury, although we did not observe increased infiltration of macrophages in the DRGs of d4T-treated rats previously (Huang et al., 2013). Proteomic analysis in the sural nerve of d4T-treated rats has revealed the down-regulation of microtubule-associated protein 1B that may be involved in the ‘dying back’ pathology (Huang et al., 2013). The spinal cord may also be involved, with increases in dorsal horn BDNF expression (Renn et al., 2011) and length-dependent reductions in CGRP and IB4 expression in the superficial lamina of the spinal dorsal horn in d4t-treated rats (Huang et al., 2013).

## 5. Conclusion

This study has revealed that there are distinct differences in the neurochemical responses to nerve damage in the DRGs in two different types of nerve damage that are both associated with neuropathic pain, highlighting the heterogeneity of the mechanisms of neuropathic pain from different aetiologies. Evidence from clinical studies have shown that neuropathic pain patients can exhibit varying pain symptoms and sensory profiles revealed by quantitative sensory testing, even those with similar aetiologies, revealing the heterogeneity of neuropathic pain and the likelihood of various pain mechanisms (Maier et al., 2010; Baron et al., 2012). This proposes the prospect that neuropathic pain research can be approached in terms of its presumed mechanisms, irrespective of aetiology, and if those mechanisms are targeted specifically, it may allow an optimum treatment regime that could lead to better neuropathic pain management (Finnerup et al., 2010).

## Supporting Information

Appendix S1 Additional methods and results to demonstrate heterogeneous DRG responses in two distinct neuropathies. **Figure S1.** Time course of TNT-associated mechanical hypersensitivity. The PWT to punctate mechanical stimuli was measured in different groups of animals examined at 1, 7, 14, 28 and 43 days following TNT and sham surgery. Baseline PWTs were determined for all operated animals prior to surgery. Data are presented as mean ± SEM. ***p* < 0.01 and **p* < 0.05 versus respective baseline levels using a Student's paired *t*-test. **Figure S2.** Time course of d4T-associated mechanical hypersensitivity. The PWT to punctate mechanical stimuli were measured in left and right hind limbs at the various time point post d4T/saline injections. Data are presented as mean ± SEM. ***p* < 0.01 and **p* < 0.05 versus respective baseline levels using a Student's paired *t*-test. **Figure S3.** Representative images of NPY immunoreactivity. The left panel is for NPY expression in ipsilateral L5 DRGs at 1 and 14 days post-TNT injury and at 14 days post-sham surgery. The right panel is for NPY expression in the left L5 DRGs of d4T/saline-treated rats at 7 days post first injection, and in the L5 DRGs of naïve animals. Sections were co-labelled with peripherin and NF-200 to identify NPY immunoreactivity in distinct populations of DRG neurons. Images were captured at ×20 objective magnification. Arrows indicate co-localization of immunolabelling. Scale bar = 50 μm. **Figure S4.** Representative images of galanin immunoreactivity. The left panel is for galanin expression in ipsilateral L5 DRGs at 1 and 14 days post-TNT injury and at 14 days post-sham surgery. The right panel is for galanin expression in the left L5 DRGs of d4T/saline-treated rats at 7 days post first injection, and in the L5 DRGs of naïve animals. Sections were co-labelled with peripherin and NF-200 to identify galanin immunoreactivity in distinct populations of DRG neurons. Images were captured at ×20 objective magnification. Arrows indicate co-localization of immunolabelling. Scale bar = 50 μm. **Table S1.** Major domains of good laboratory practice to minimize the effects of experimental bias on both hindpaw mechanical sensory testing and immunohistochemical analysis. **Table S2.** Mean percentage immunoreactivity and mean cell area (μm^2^) of ATF-3, GAP-43, NPY and galanin in NF-200 and peripherin-immunoreactive cells in naïve L5 DRGs. Data are presented as mean ± SEM. The numbers of ATF-3, GAP-43, NPY and galanin immunoreactivity cells out of the total number of peripherin/NF-200-immunoreactive cells are indicated in brackets (n/a, not applicable as no data was available).
