# Supplementary material for: Heterogeneous responses of dorsal root ganglion neurons in neuropathies induced by peripheral nerve trauma and the antiretroviral drug stavudine
Source: Eur J Pain. 2014 Jul 29;19(2):236–45. doi: 10.1002/ejp.541 (PMC4312904; doi:10.1002/ejp.541)
Supplement: Appendix S1 — Additional methods and results to demonstrate heterogeneous DRG responses in two distinct neuropathies. Figure S1. Time course of TNT-associated mechanical hypersensitivity. The PWT to punctate mechanical stimuli was measured in different groups of animals examined at 1, 7, 14, 28 and 43 days following TNT and sham surgery. Baseline PWTs were determined for all operated animals prior to surgery. Data are presented as mean ± SEM. **p < 0.01 and *p < 0.05 versus respective baseline levels using a Student's paired t-test. Figure S2. Time course of d4T-associated mechanical hypersensitivity. The PWT to punctate mechanical stimuli were measured in left and right hind limbs at the various time point post d4T/saline injections. Data are presented as mean ± SEM. **p < 0.01 and *p < 0.05 versus respective baseline levels using a Student's paired t-test. Figure S3. Representative images of NPY immunoreactivity. The left panel is for NPY expression in ipsilateral L5 DRGs at 1 and 14 days post-TNT injury and at 14 days post-sham surgery. The right panel is for NPY expression in the left L5 DRGs of d4T/saline-treated rats at 7 days post first injection, and in the L5 DRGs of naïve animals. Sections were co-labelled with peripherin and NF-200 to identify NPY immunoreactivity in distinct populations of DRG neurons. Images were captured at ×20 objective magnification. Arrows indicate co-localization of immunolabelling. Scale bar = 50 μm. Figure S4. Representative images of galanin immunoreactivity. The left panel is for galanin expression in ipsilateral L5 DRGs at 1 and 14 days post-TNT injury and at 14 days post-sham surgery. The right panel is for galanin expression in the left L5 DRGs of d4T/saline-treated rats at 7 days post first injection, and in the L5 DRGs of naïve animals. Sections were co-labelled with peripherin and NF-200 to identify galanin immunoreactivity in distinct populations of DRG neurons. Images were captured at ×20 objective magnification. Arrows indicate co-l [file ejp0019-0236-sd1.doc]

Heterogeneous responses of dorsal root ganglion neurons in neuropathies induced by peripheral nerve trauma and the antiretroviral drug stavudine

Supplementary Materials

**1. Additional methods:**

## 1.1) Tibial Nerve Transection (TNT) surgery

TNT surgery was performed under general anaesthesia with 2-3% isoflurane (Abbott, UK) in O_2_ (1.5L/min), using an aseptic technique (Andrews et al., 2012). A skin incision was made over and parallel to the femur in the left hind limb. An incision was made along the fascial plane between the gluteus superficialis and biceps femoris muscles to expose the trifurcation of the sciatic nerve. The tibial nerve was identified and then ligated with 7/0 silk suture (Ethicon, UK) at 2-3mm from where the tibial nerve branches from the sciatic nerve. A subsequent ligature was made ~2mm caudally, prior to the subsequent branching of the tibial nerve and the nerve transected between the two ligatures. The nerve was gently placed back and the muscle and skin closed with 4/0 silk suture (Ethicon, UK). Sham surgery was performed by exposing the tibial nerve without ligation and transection. Postoperative analgesia consisted of subcutaneous injection of 0.5% bupivacaine (Antigen Pharmaceuticals, Ireland) to the wound site followed by intraperitoneal administration of Carprofen (20%, 0.1ml/200g; Pfizer, UK) 2-4hr later.

## 1.2) Procedure for administration of d4T

Under 2-3% isoflurane anaesthesia (Abbott, UK) in O_2_ (1.5L/min), rats received two 0.5ml intravenous injections (separated by 4 days) of d4T (50mg/kg in 0.9% sterile saline; gift from Pfizer) or 0.9% sterile saline into the tail vein (Huang et al., 2013). Although patients are generally administered d4T orally, previous studies have shown that both daily oral gavage and a single intravenous administration routes produce similar nocifensive behavioural profiles in rats (Joseph et al., 2004). Thus, we opted for the intravenous route in order to minimise the handling stress to animals associated with oral gavage.

**1.3) Rationale for investigating markers (ATF-3, GAP-43, NPY, and galanin):**

Activating transcription factor-3 (ATF-3) is induced following nerve trauma injury, and is commonly used as a marker of nerve damage. ATF-3 was chosen for the study since as a marker of nerve damage, its expression following tibial nerve transaction (TNT) could be compared to the non-traumatic d4T model of peripheral nerve damage. In addition the temporal expression of ATF-3 has not been examined in d4T-treated rats where there is clear evidence of nerve damage with the dying back of peripheral nerve fibres. Interestingly, ATF-3 expression has been linked to the ability of injured peripheral neurons to regenerate (Flatters and Bennett 2006). The expression of ATF-3 would indicate such a process occurring. In order to further assess evidence of nerve regeneration we examined the expression of growth associated protein-43 (GAP-43). Following traumatic peripheral nerve injury, damaged neurons have the capacity to regenerate and this has been associated with the robust expression of GAP-43. The temporal expression of GAP-43 in DRG of d4T-treated rats additionally has not been previously investigated and therefore was selected for investigation.

Galanin and neuropeptide Y (NPY) were selected for investigation, as they are commonly dysregulated proteins following nerve trauma injury. The anatomical location of galanin and its receptors in DRG and the superficial dorsal horn is of interest and suggests that galanin may also participate in the regulation of nociceptive transmission and thus pain (Hokfelt et al., 1987). NPY has also been implicated in the regulating nociception. NPY and its receptors are located in regions of the spinal cord that are associated with nociceptive transmission such as the superficial lamina of the dorsal horn (Gibson et al., 1984; Ma and Bisby 1998)**.** The intrathecal administration of NPY attenuates behavioural responses to noxious heat in normal animals (Hua et al., 1991) and inflammatory model of pain (Taiwo and Taylor 2002), and mechanical and cold hypersensitivity in the SNI peripheral nerve injury model (Intondi et al., 2008). The knockout of the NPY Y1 receptor induces a hypersensitive state to mechanical stimuli (Naveilhan et al., 2001; Shi et al., 2006). The histological, transgenic, pharmacological, and behavioural studies together support the notion that NPY and galanin play a role in regulating nociceptive transmission as well as nerve injury, and perhaps neuropathic pain. The relevance and the temporal expression patterns of these proteins in the non-traumatic d4T drug induced nerve damage model have not been fully evaluated.

Supplementary Table 1. Major domains of good laboratory practice to minimize the effects of experimental bias on both hindpaw mechanical sensory testing and immunohistochemical analysis.

|  | **Description of procedures** |
| --- | --- |
| Sample size calculation | - A sample size analysis was performed based on previous studies within our group using the Spinal nerve transection (SNT) (Hasnie et al., 2007) and ddC (Wallace et al., 2007) models**.** - Sample sizes calculated using SigmaStat Version 3.5 (desired power=0.8 and alpha=0.05). - TNT sample size calculation: The minimum detectable difference in the mean PWT in SNT animals was 25g and the standard deviation was 8g (14 days post injury). A sample size of 4 animals was calculated per group. - D4T sample size calculation: The minimum detectable difference in the mean PWT in ddC animals was 16g and the standard deviation was 5g (14 days post injury). A sample size of 4 animals was calculated per group. |
| Inclusion and exclusion criteria | - TNT operated rats that did not exhibit transection of the tibial nerve or transection of an incorrect nerve (identified upon post mortem) were excluded from all results. - TNT operated rats were planned to be excluded from analysis if they did not display a >30% reduction in ipsilateral paw withdrawal threshold (relative to pre-surgery responses, except at post injury day one). |
| Randomization | - For both TNT and d4T experiments, treatments were determined by picking cage numbers out of a hat and the order of treatment for each cage was determined using a computerized random sequence generator (www.random.org). - For image analysis, slide labels were masked using tape and subsequently numbered, and the order of analysis determined using the computerized random sequence generator. |
| Allocation concealment | - The person creating the model (i.e. injection of d4T/saline solution, or performing TNT/sham surgeries) was unaware of the allocation to treatment group. |
| Reporting of animal exclusions | - Any rat showing hunched posture, a marked behavioural change, exudates around wound or sensitivity to palpitation on handling that could be attributable to surgery, the drug, the dosing procedure, infection resulting from surgery or otherwise, was excluded. - Any rat with significant surgical complications, or whose general health deteriorates, was excluded. - Limited motor dysfunction or autotomy may occur after TNT injury, but very rarely. If a persistent motor impairment whereby the animal is prevented from reaching food and water was observed then the animal was excluded. - Similarly, if autotomy of more than one digit was observed then the animals were excluded. - The details of the number of excluded animals and the reason for exclusion are stated in the results section. |
| Experimental outcomes | - The primary outcome measures are hindpaw reflex withdrawal responses to static mechanical stimuli and the expression of neurochemical markers: ATF-3, GAP-43, NPY, and galanin in L5 DRGs. - Hindpaw sensory testing and image analysis were performed by a scientist blinded to surgical status of the rats. |

**2. Additional results:**

Supplementary Table 2. Mean percentage immunoreactivity and mean cell area (μm^2^) of ATF-3, GAP-43, NPY, and galanin in NF-200 and peripherin immunoreactive cells in naïve L5 DRGs. Data are presented as mean±SEM. The numbers of ATF-3, GAP-43, NPY, and galanin immunoreactivity cells out of the total number of peripherin/NF-200 immunoreactive cells are indicated in brackets (n/a=not applicable as no data was available).

| **Naïve L5 DRG** | | |
| --- | --- | --- |
| **Markers** | **Mean % immunoreactivity** | **Mean cell area μm^2^** |
| **ATF-3**  Peripherin  NF-200 | 0  0 | n/a (0/632)  n/a (0/326) |
| **GAP-43**  Peripherin  NF-200 | 0  1.73±1.73 | n/a (0/833)  997.5±100.5 (7/285) |
| **NPY**  Peripherin  NF-200 | 0  0.28±0.28 | n/a (0/996)  1609.1 (1/268) |
| **Galanin**  Peripherin  NF-200 | 0.56±0.56  0 | 477.2±33.0 (6/893)  n/a (0/345) |

Supplementary Fig. 1


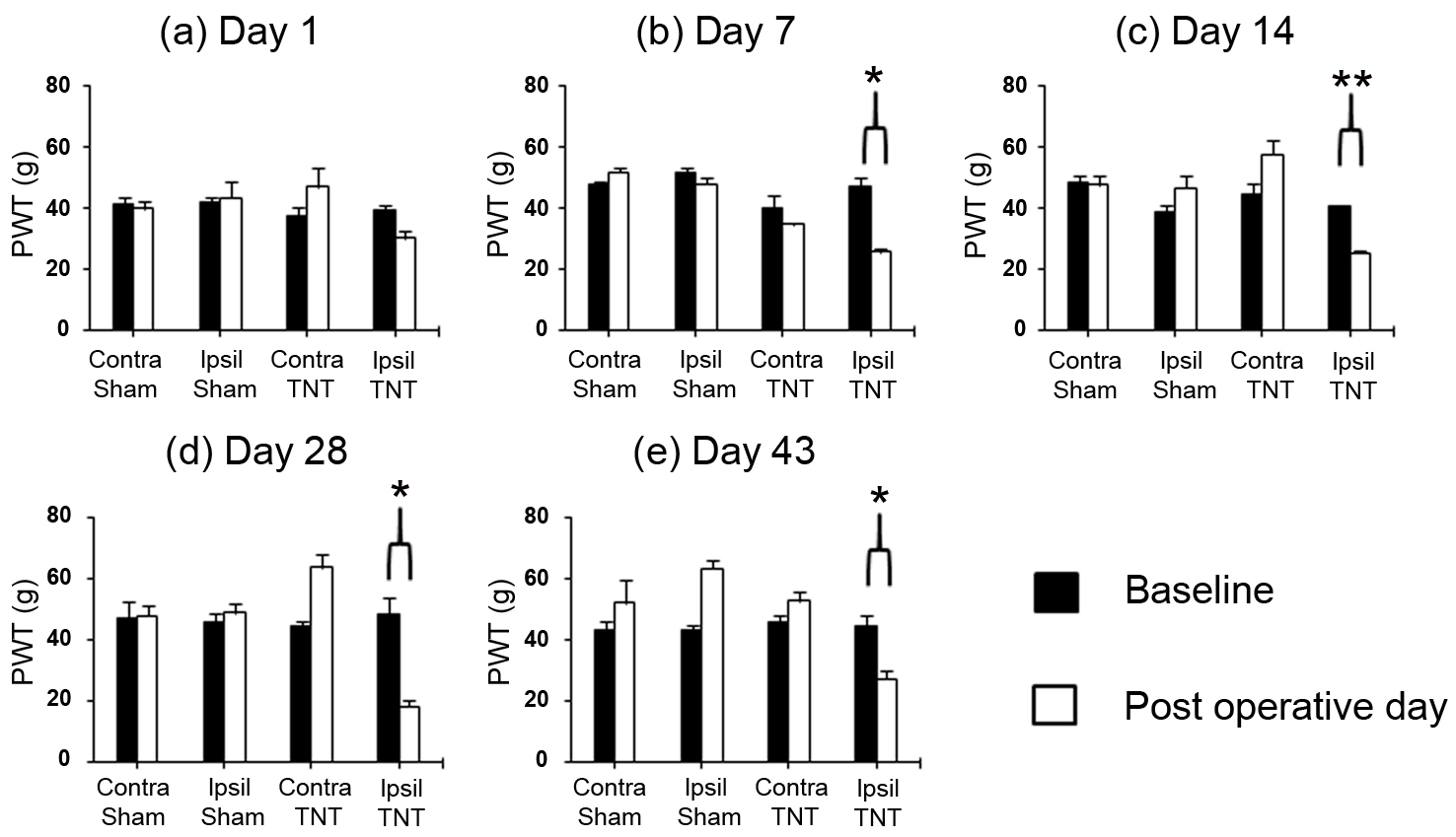


**Supplementary Fig. 1.** Time course of TNT-associated mechanical hypersensitivity. The PWT to punctate mechanical stimuli was measured in different groups of animals examined at 1, 7, 14, 28, and 43 days following TNT and sham surgery. Baseline PWT were determined for all operated animals prior to surgery. Data are presented as mean±SEM. **P<0.01 and *P<0.05 vs. respective baseline levels using a Student’s paired t-test.

Supplementary Fig. 2


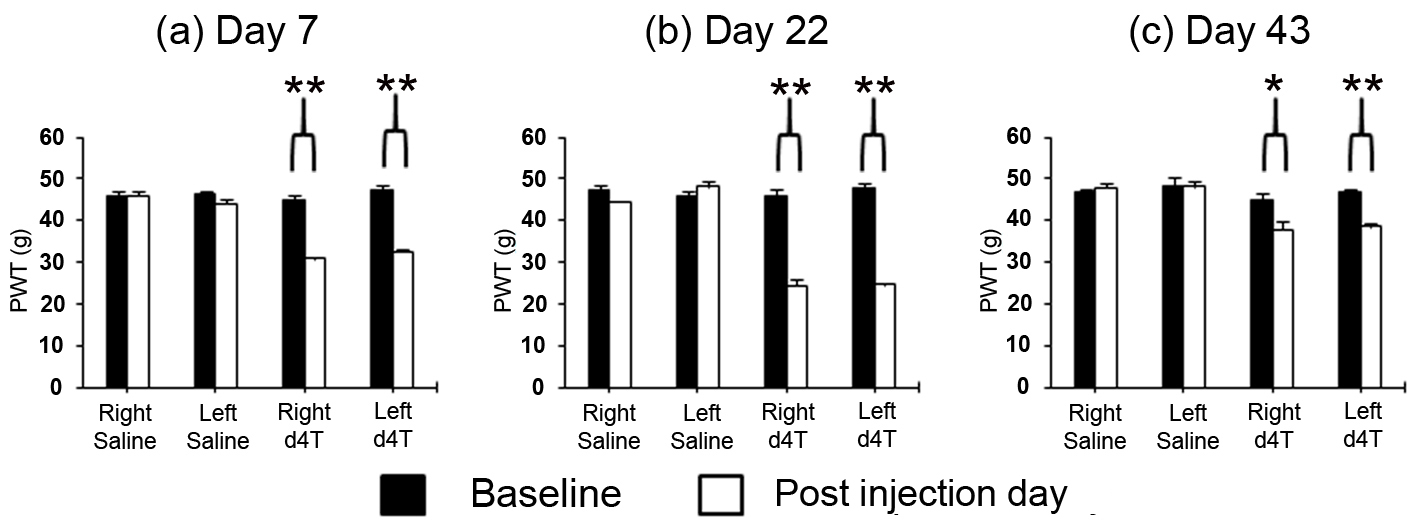


Supplementary Fig. 2. Time course of d4T-associated mechanical hypersensitivity. The PWT to punctate mechanical stimuli were measured in left and right hind limbs at the various time point post d4T/saline injections. Data are presented as mean±SEM. ***P*<0.01 and **P*<0.05 vs. respective baseline levels using a Student’s paired t-test.

Supplementary Fig. 3


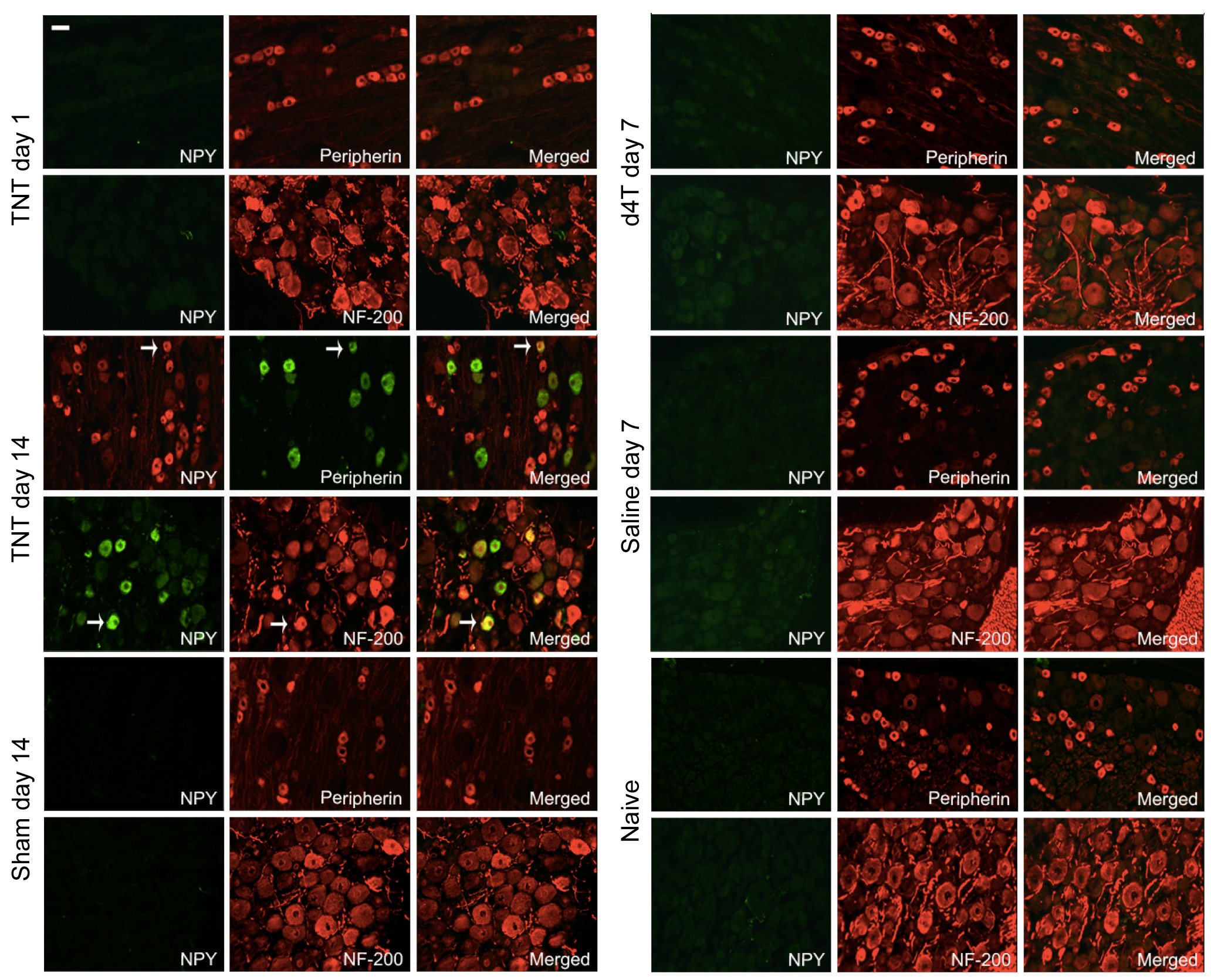


**Supplementary Fig. 3.** Representative images of NPY immunoreactivity. The left panel is for NPY expression in ipsilateral L5 DRGs at 1 and 14 days post TNT injury and at 14 days post sham surgery. The right panel is for NPY expression in the left L5 DRGs of d4T/saline-treated rats at 7 days post first injection, and in the L5 DRGs of naïve animals. Sections were co-labelled with peripherin and NF-200 to identify NPY immunoreactivity in distinct populations of DRG neurons. Images were captured at x20 objective magnification. Arrows indicate co-localisation of immunolabelling. Scale bar=50μm.

Supplementary Fig. 4


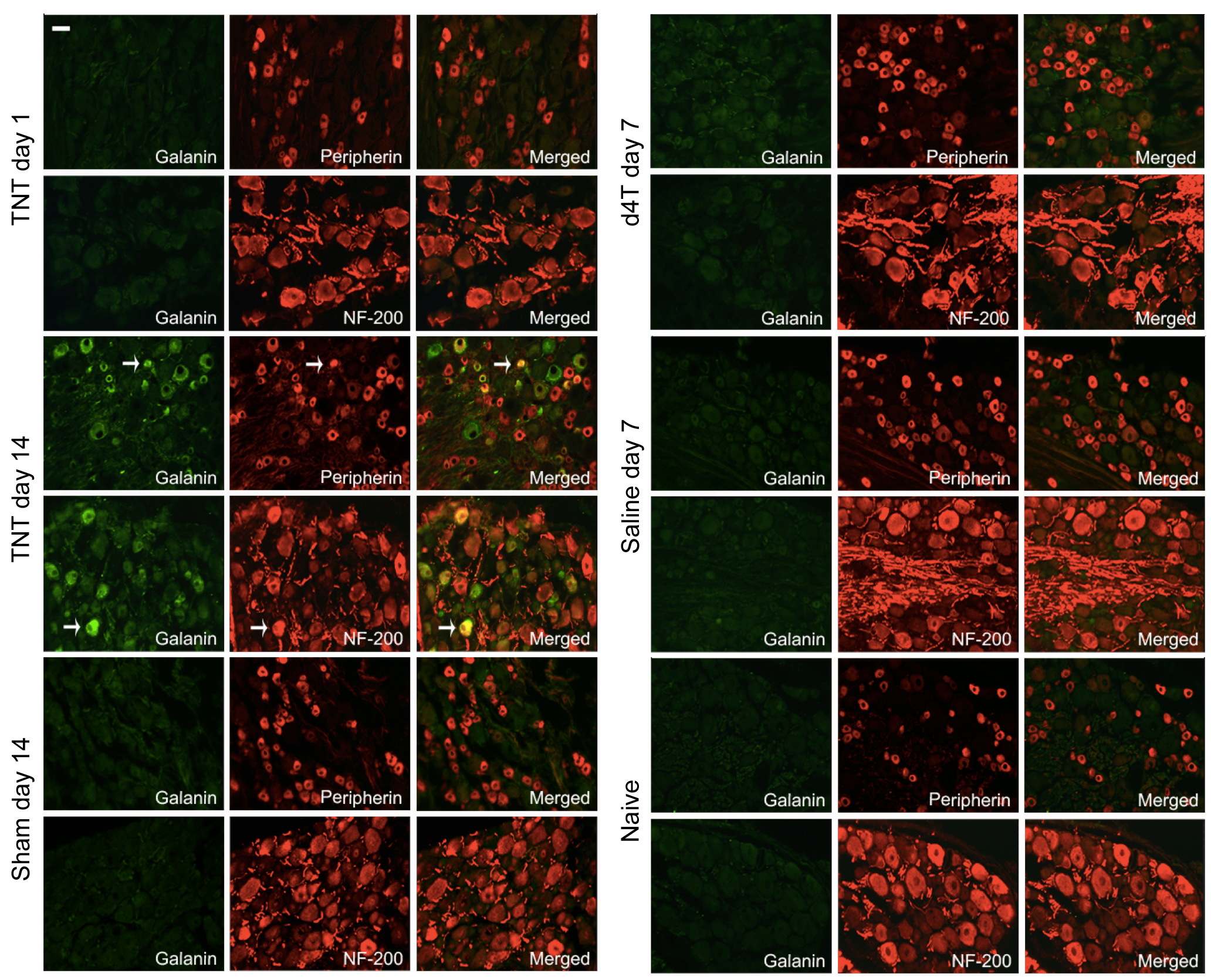


**Supplementary Fig. 4.** Representative images of galanin immunoreactivity. The left panel is for galanin expression in ipsilateral L5 DRGs at 1 and 14 days post TNT injury and at 14 days post sham surgery. The right panel is for galanin expression in the left L5 DRGs of d4T/saline-treated rats at 7 days post first injection, and in the L5 DRGs of naïve animals. Sections were co-labelled with peripherin and NF-200 to identify galanin immunoreactivity in distinct populations of DRG neurons. Images were captured at x20 objective magnification. Arrows indicate co-localisation of immunolabelling. Scale bar=50μm.

Reference

Andrews, N., Legg, E., Lisak, D., Issop, Y., Richardson, D., Harper, S., Pheby, T., Huang, W., Burgess, G., Machin, I., Rice, A.S. (2012). Spontaneous burrowing behaviour in the rat is reduced by peripheral nerve injury or inflammation associated pain. *EurJ Pain* **16**,485-495.

Flatters, S.J. and Bennett, G.J. (2006). Studies of peripheral sensory nerves in paclitaxel-induced painful peripheral neuropathy: evidence for mitochondrial dysfunction. *Pain* **122**,245-257.

Gibson, S.J., Polak, J.M., Allen, J.M., Adrian, T.E., Kelly, J.S., Bloom, S.R. (1984). The distribution and origin of a novel brain peptide, neuropeptide Y, in the spinal cord of several mammals. *J Comp Neurol* **227**,78-91.

Hasnie, F.S., Breuer, J., Parker, S., Wallace, V., Blackbeard, J., Lever, I., Kinchington, P.R., Dickenson, A.H., Pheby, T., Rice, A.S.C. (2007). Further characterization of a rat model of varicella zoster virus-associated pain: Relationship between mechanical hypersensitivity and anxiety-related behavior, and the influence of analgesic drugs. *Neuroscience* **144**,1495-1508.

Hokfelt, T., Wiesenfeld-Hallin, Z., Villar, M., Melander, T. (1987). Increase of galanin-like immunoreactivity in rat dorsal root ganglion cells after peripheral axotomy. *Neurosci Lett* **83**,217-220.

Hua, X.Y., Boublik, J.H., Spicer, M.A., Rivier, J.E., Brown, M.R., Yaksh, T.L. (1991). The antinociceptive effects of spinally administered neuropeptide Y in the rat: systematic studies on structure-activity relationship. *J Pharmacol Exp Ther* **258**,243-248.

Huang, W., Calvo, M., Karu, K., Olausen, H.R., Bathgate, G., Okuse, K., Bennett, D.L.H., Rice, A.S.C. (2013). A clinically relevant rodent model of the HIV antiretroviral drug stavudine induced painful peripheral neuropathy. *Pain* **154**,560-575.

Intondi, A.B., Dahlgren, M.N., Eilers, M.A., Taylor, B.K. (2008). Intrathecal neuropeptide Y reduces behavioral and molecular markers of inflammatory or neuropathic pain. *Pain* **137**,352-365.

Joseph, E.K., Chen, X., Khasar, S.G., Levine, J.D. (2004). Novel mechanism of enhanced nociception in a model of AIDS therapy-induced painful peripheral neuropathy in the rat. *Pain* **107**,147-158.

Ma, W. and Bisby, M.A. (1998). Partial and complete sciatic nerve injuries induce similar increases of neuropeptide Y and vasoactive intestinal peptide immunoreactivities in primary sensory neurons and their central projections. *Neuroscience* **86**,1217-1234.

Naveilhan, P., Hassani, H., Lucas, G., Blakeman, K.H., Hao, J.X., Xu, X.J., Wiesenfeld-Hallin, Z., Thoren, P., Ernfors, P. (2001). Reduced antinociception and plasma extravasation in mice lacking a neuropeptide Y receptor. *Nature* **409**,513-517.

Shi, T.J.S., Li, J., Dahlstr”m, A., Theodorsson, E., Ceccatelli, S., Decosterd, I., Pedrazzini, T., H”kfelt, T. (2006). Deletion of the neuropeptide Y Y1 receptor affects pain sensitivity, neuropeptide transport and expression, and dorsal root ganglion neuron numbers. *Neuroscience* **140**,293-304.

Taiwo, O.B. and Taylor, B.K. (2002). Antihyperalgesic effects of intrathecal neuropeptide Y during inflammation are mediated by Y1 receptors. *Pain* **96**,353-363.

Wallace, V.C., Blackbeard, J., Segerdahl, A.R., Hasnie, F., Pheby, T., McMahon, S.B., Rice, A.S. (2007). Characterization of rodent models of HIV-gp120 and anti-retroviral-associated neuropathic pain. *Brain* **130**,2688-2702.
